# The role of gut microbiota in autoimmune disease progression and therapy: a comprehensive synthesis

**DOI:** 10.3389/frmbi.2025.1553243

**Published:** 2025-05-16

**Authors:** Mohammad Adawi

**Affiliations:** Laniado MC, Ariel University, General Health Services, Natanya, Israel

**Keywords:** gut microbiome, autoimmune diseases, dysbiosis, immune regulation, microbiome-targeted therapy

## Abstract

Autoimmune diseases arise from the immune system’s dysregulated attack on the body’s own tissues, influenced by a complex interplay of genetics, environment, and the microbiome. This comprehensive review and meta-analysis examines the dynamic relationship between gut microbiota and autoimmune diseases, highlighting their role in disease onset, progression, and potential therapeutic interventions. Emerging evidence underscores the bidirectional interactions between microbiota and immune pathways, particularly through mechanisms like mucosal immune modulation and regulatory T-cell activity. Microbiota dysbiosis, characterized by altered diversity and function, is consistently associated with autoimmune conditions such as rheumatoid arthritis, multiple sclerosis, and type 1 diabetes. The review identifies critical microbiota-driven factors, including antigenic mimicry and inflammatory signaling pathways that disrupt immune tolerance and exacerbate autoimmunity. Meta-analysis findings reveal a consistent reduction in microbial diversity across autoimmune diseases, emphasizing the role of specific taxa and their metabolites in influencing disease severity and immune responses. Therapeutic strategies, such as probiotics, prebiotics, and microbiome-targeted interventions, offer promising avenues to restore microbiome balance and mitigate autoimmune inflammation. Despite significant advances, challenges in methodology, limited longitudinal studies, and heterogeneity in results highlight the need for standardized research protocols and larger, well-controlled clinical trials. Future studies should prioritize personalized approaches to microbiome modulation, integrating dietary, genetic, and environmental factors to improve disease management and prevention. This work consolidates current knowledge, providing a framework for future research and clinical applications in the field of microbiome-autoimmune interactions.

## 1 Introduction

Autoimmune diseases are conditions in which the immune system, which typically fights off foreign invaders, turns its destructive power against the body’s own cells or tissues. This aberrant response can result from the interplay between genetics, environment, and gut microbiota. The gut microbiota are the microorganisms living under homeostasis in our digestive tracts, including bacteria, viruses, fungi, and protozoa. Exposing the gut to foreign environmental antigens should result in either an inactivating mucosal immune response or immune tolerance. However, a disruption to the gut microbiome or impaired mucosal barrier can allow entering antigens to interface with the mucosal immune system in a pro-inflammatory way. Over time, this dysfunction can trigger a systemic immune response, driving pathogenic immune cells and autoimmune disease. We, therefore, undertook a comprehensive review explore the potential relationships between the gut microbiome and various autoimmune diseases. We found experimental evidence that the probable relationships become apparent through modulation of microbiota communities or their individual members, which has led to a demonstrable impact on autoimmunity in both human and rodent gut models of disease and is, therefore, now a widely accepted concept. A class of T-helper 17 cells was identified as the mucosal pathogen at the forefront of this mechanism. This cell population is positively correlated with disease severity. We further discussed the potential functional roles of vaccine-induced responses to the microbiome in autoimmune disease abrogation and treatment. As such, the gut microbiome has potential diagnostic significance to predict disease progression (Belkaid and Hand, 2014; De Luca and Shoenfeld, 2018).

### 1.1 Background and rationale

Autoimmune and autoinflammatory diseases (ADs and AIDs) represent a significant health burden. The microbiome, which encompasses the microbiota, the host genome, and environmental factors, creates an environment that influences immune responses. ADs and AIDs are linked to the microbiome through factors such as the gut-brain axis and the determination of predisposition. With the increasing interest in the microbiome, much research has focused on the pathology of each individual disease, yet this has yielded controversial and inconsistent results. Integrating each disease from the perspective of genetics and clinical phenotypes is necessary, systematic review can help to illustrate the changes of the microbiome in the different paths and phenomena within human microbial diseases. The mechanisms of the relationship between the microbiome and autoimmunity can also be demonstrated through the literature. We collect up-to-date related research and select the available studies by filtering criteria and searching on reputable global articles. We illustrate the features and species affecting microbial diversity in ADs through alpha and beta diversity analyses and create a pipeline that can demonstrate the relationship among diseases (Zhang et al., 2023).

### 1.2 Scope and objectives

The review and original analysis herein undertaken help provide a connection between autoimmune diseases and antibiotic use, as well as the digestion of specific immune-modulating dietary components, such as fibers and sweeteners. The primary objective of the review was to develop a comprehensive overview of all current mapping studies on the microbiome of autoimmune diseases in order to establish relationships among them and find common trends that will ultimately be able to explain the current results trend for both the microbiome’s impact on autoimmune diseases and autoimmune disease effects on the microbiome. A second perspective of the work is to provide a mapping framework for all future studies on this topic and develop a way of tracking research on this subject. The third objective of the work is to show that disparities exist between studies and that more publications on microbiome and autoimmunity disease research are needed to provide sufficient information to develop a deeper understanding of microbiome contributions to autoimmune disease development and progression. Lastly, it is important to identify the haphazard use of the rational manipulation of the microbiome that can help prevent or cure new and even existing illnesses. To achieve this result, this review evaluates the significant methodical disparities among microbiome studies and how they affect the results of the study and how the relative exiguity of strategic treatment recommendations can be adopted from up-to-date research findings. Furthermore, we explore pertinent proof gathered in the progression of some of the most common ailments, demonstrating that other microbiome manipulation techniques are corroborated and that significant scientific trials are missing. To fulfill these aims, we entice studies from a broad range of sectors and, focused on current knowledge, employ an interdisciplinary approach to merge all relevant sources. Cutbacks in internal microbiome multifaceted diversity are associated with serious diseases, including autoimmunity. In this assessment, we have previously expanded work to benefit the public; particularly, approaches concerned with autoimmune diseases incorporate joint information (Wang et al., 2024).

## 2 Understanding the microbiome

While some scientists and laypersons have known about the existence of commensal bacteria in the human gut and on the skin for decades, many breakthrough discoveries in the understanding of how these species contribute to host biology and environmental ecology have occurred within the last 20 to 30 years, enabled by innovations in bioinformatics and supported by the rapidly decreasing costs of whole-genome shotgun sequencing. These species, individually termed microbiota, interact with the host and environmental pathogens to provide a number of essential functions that the host lacks, including metabolism of complex carbohydrates, fermentation of otherwise indigestible substrates, and metabolism of plant polysaccharides, which together produce short-chain fatty acids, such as butyrate, acetate, and propionate, that can contribute upward of 10% of a human’s total caloric intake. The bacterial residents of the gastrointestinal lumen also play critical roles in resistance to exogenous pathogens by facilitating the development of the host’s mucosal immunity. Animal models suggest that gut commensal bacteria play an important role in modulating a variety of autoimmune responses, including neurological autoimmunity, thyroiditis, and gastritis. Indeed, the presence of many of these species in the infant gut influences early development of the immune system and etiological risk for autoimmune diseases. Moreover, maintenance of a healthy colonic microbiome influences the chronic inflammation and disease progression homeostasis in patients with many autoimmune and autoinflammatory diseases. Homeostatic autoreactivity in these organs is shaped directly by the presence of elevated pro-inflammatory metabolites, especially in the gut and oral microbiome. High levels of systemic lipopolysaccharides and autoantigens are, consequently, prone to breach the normally protective gut lumen barrier, leading to chronic inflammation and breakdown in oral tolerance that can contribute to the genesis and persistence of autoimmune disease (Berg et al., 2020).

### 2.1 Definition and composition

The gastrointestinal microbiome, also termed microbiota, is typically described as a distinct unit of bacterial communities. In addition to prokaryotic bacteria, the biliary microbiome also consists of fungal and eukaryotic components connected to liver diseases. Nevertheless, the gut microbiome is composed of thousands of different taxa, including bacteria, viruses, and fungi, incorporated in a specific environment of host-fed, whole habitat bacterial complexes. Moreover, metatranscriptomics and metaproteomics have indicated that functional performance is not fairly mirrored by every taxonomic inventory. Human feces can reach bacterial particle concentrations as high as 10^11 or 10^12 per gram of feces, leading to the duplication of over 10 million genes in human intestinal communities. Therefore, setting apart person-to-person genetic diversity, the coding project DNA contained by indigenous human microorganisms far surpasses the predictable gene copies found in the genome of humans alone. Indeed, the microbial gene count might be as much as 150 times as heavy as the related host genome (Gosalbes et al., 2011).

### 2.2 Role in human health

The development of the human immune system is influenced by both genetic and environmental factors. In recent years, scientific interest has been actively developing, linking the state of the human immune system with environmental influences, including microbiota and its components. Accumulated evidence suggests that the microbiota contributes to the education of the immune system. The shaping of immune-competent cells of the systemic immune system and the balance between the processes of tolerance and immune activation are not completely formed, with the education of the child’s immune system including the synthesis of cytokines that form the direction of the immune response closely linked to the presence of an intact microbiota. The first experimental evidence indicating the influence of the microbiota on human health was discovered in studies evaluating the development and functionality of the immune system. These studies concluded that the colonization of children with ‘good’ microorganisms after birth is important for both the development of resistance to infections and the prevention of inflammatory conditions. Studies assessing groups of monozygotic and dizygotic twins showed a greater similarity between monozygotes associated with a more similar structure of the microbiota and greater variability in the expression of genes associated with immunity in dizygotes. In other words, the development of an individual is characterized by the personality of immunity, which is related to the unique individual structure of the microbiota. More recent studies have described a complex relationship between microbes, immunity, and disease. The result of this balance between the two is the state of the host immune responses, with the microbiota influencing a variety of different aspects. It determines our defenses against infections and modulates our immune responses to commensal or harmful microbes, influences the development of immune tolerance at epithelial and systemic levels, shapes the profiles of different effector elements, both innate and acquired, and influences the function of various hematopoietic cells in secondary lymphoid organs and barriers, such as intestine and lung (Ahn and Hayes, 2021).

## 3 Autoimmunity: mechanisms and implications

Autoimmune diseases are an increasingly prevalent group of non-communicable disorders driven by autoimmunity, which is defined as an adaptive immune response that damages its host. Autoimmunity is a complex disorder characterized by the presence of circulating autoantibodies and autoreactive T cells against self-antigens, combined with an autoimmune response against specific organs and tissues of the body. Autoimmune diseases have a strong genetic basis; this is supported by classical twin studies, sibling studies, and segregation analyses of familial risk. However, genes do not act in isolation; non-genetic factors related to the environment, and especially the gut microbiota, provide further potential insights into the development of these diseases. The term microbiota encompasses all the genetic material within a microbiome, including bacteria, archaea, fungi, protozoa, and viruses, alongside their cohabiting commensal and sentinel immune cell partners. The microbiota exerts a protective influence on autoimmunity by promoting immune regulation, although it can also trigger a pro-inflammatory immune response, thus favoring the progression of autoimmune responses and the development of inflammatory and autoimmune diseases. The exact nature of the microbiome’s influence on autoimmunity is likely to be multifactorial. The implications of the cross talk between the microbiome and autoimmunity are widespread and prompt us to consider how to best maintain a harmonious relationship with the microbiome to promote health and well-being throughout our lives. Data from humanized mice showed that the ability to develop immune tolerance is lost in response to colonization and the interaction with environmental bacteria—a process described as the hygiene deficit. In these mice, the development of autoimmune diseases such as type 1 diabetes or other inflammatory diseases became faster than in specific pathogen-free animals. In sum, certain environmental bacteria or other microorganisms might protect the host from the development of chronic mucosal diseases by acting as natural adjuvants or immunostimulants that induce a tonic volumetric homeostasis of the immune system (Miller, 2022).

### 3.1 Definition and examples

The concept of the “microbiome” refers to all microbes, their genetic material, or their products within a specific compartment or in the organism. The most studied microbiome is the gut microbiome, but there are other microbiomes such as the lung, skin, and oral microbiomes. Like human genetic data, the microbiome is incredibly diverse and variable. There is great variability in microbiome composition between individuals and body sites within individuals. The microbiome is influenced by host genetics, the local environment, health status, and external interventions such as antibiotics, immunosuppressants, and anti-inflammatories (Hou et al., 2022).

### 3.2 Pathogenesis and triggers

The Gut Barrier and Autoimmunity Mechanisms that are responsible for the development of autoimmune diseases often feature damage to bodily tissues due to T-cell mediated auto aggression. In the case of type 1 diabetes, for example, the major antigen is expressed on the islet beta-cell surface. Once infiltrated, the immune system can differentiate these cells as pathologically changed and launch an immune response against them. The natural history of such disease development has a number of clear steps. First, the immune system of susceptible individuals is sensitized to self-antigens due to exposure to various triggers. Second, antigen-specific, autoreactive T cells escape thymic negative selection and are activated by environmental triggers, moving the disease into the clinically silent phase. Finally, the autoimmune reaction that leads to disease manifests and progresses. The triggers that initiate autoantigen self-activation in order to form clonal proliferating immune cells and break self-tolerance have historically been divided into interplaying genetic and environmental factors. The disease itself or a genetic predisposition to some pathologies could sometimes be introduced into a healthy body by the transplantation of hematopoietic stem cells carrying apoptotic bodies formed in the blood of diseased subjects. This process has been called disorder transfer through blood stem cell transplantation. These data, therefore, strongly support the hypothesis that immune cells committed to auto aggression can enter a quiescent state, and this trait could be inherited by daughter cell generations. After that, disruption in peripheral control mechanisms could provoke disease onset. Despite the fact that among autoimmune diseases, there are both candidates for thymic presentation and ectopic lymphoid organs, there is no clear proof of the general role of the thymus in controlling autoimmune reactions (Burrack et al., 2017).

## 4 The interplay between microbiome and autoimmunity

The relationship between the gastrointestinal microbiome and autoimmunity may be bidirectional in nature. Studies to date have indeed shown that the human microbiome is influenced by genetic, nutritional, and environmental factors, including alterations affected by medications. The impact of diet and environmental exposure, including living with pets and geographical location, on the human microbiota and on the immune system in health and disease has been well characterized. In turn, commensal microbes are critical for the development and maintenance of the host immune system by enhancing innate pattern recognition receptor expression, maintaining the recruitment of immunosuppressive cells such as regulatory T cells, and protecting the host against infection. Children and adults with autoimmune diseases are at risk for expression of the disease phenotype given the potential susceptibility of their microbiota. In animal models, it has been demonstrated that the gut microbiota can direct not only the frequency, function, and phenotypic characteristics of conventional Tregs in multiple mouse strains but can also support the differentiation of pTregs and the programming of pTregs. Tregs are present in the thymus of germ-free mice, suggesting that the gut microbiota is required for their generation in the thymus rather than their circulation from the thymus into the periphery. Given the influence of the microbiome on Tregs, the possibility that segmented filamentous bacteria or other luminal microfloral clusters may survive or migrate extra-intestinally to cause an untoward Treg response in a specific genetic host remains a tantalizing possibility. Thus, given the well-characterized importance of Tregs in controlling T-cell-dependent autoimmunity, commensal bacteria have been hypothesized to likewise modulate autoimmune diseases. It is also important to consider that these concepts may be subject to alternative explanations (De Luca and Shoenfeld, 2018; Hou et al., 2022).

### 4.1 Direct interactions

Several direct interactions have been described between the microbiome and the host immune system when a trigger like a pathogen infects the host. For example, lactate produced by Lactobacillus has immunosuppressive effects during growth. Microbe ligands also influence APCs upon TCR triggering and promote Th activation. Components of viruses, bacteria, fungi, and protozoans can directly lead to cross-reactive responses with peptides of self-antigens, increasing autoimmunity. This leads to an equilibrium in the immune system where innate and adaptive immune responses against commensals are accompanied by an induction of regulatory mechanisms that avoid immunopathology induction.

In the classical concept of a commensal lymphoid trigger, microbes induce Th17 and regulatory T cell responses that influence inflammation and homeostasis in parallel with T permeating before reported inflammation initiation. Microbes have epigenetic effects on genes that regulate the immune response, such as TLR and NOD2. Some bacteria can also produce SCFAs that can act to modulate signals in the immune system, which has important effects on inflammation and host adaptation. It is important to point out that Eubacterium limosum is capable of regulating Th/T CD4+ T ratios by expanding regulatory T cells. The species also produces a key ligand for the TCR and it is able to induce Th17 and CD4+ responses that contribute to autoimmunity if this colitogenic bacterium colonizes the gut late in life (Wiertsema et al., 2021).

### 4.2 Indirect effects

Recent evidence suggests that both gut dysbiosis and a disrupted oral microbiota are key players involved in the etiology of autoimmune diseases. Since the microbiome is capable of modifying both the innate and adaptive immune systems, indirect effects of the microbiome on autoimmune diseases are often observed. Therefore, the microbiome indirectly affects host immune response homeostasis through the mucosal immune system (Shaheen et al., 2022).

## 5 Methodology and literature search strategy

A literature extraction was conducted on various databases for peer-reviewed conditions employing thyroid, connective tissue and intestines. A focused search in journals of immunology, immunotherapy, microbiome, autoimmunity, inflammation, and allergy was conducted, respectively (Shi et al., 2017). A comprehensive systematic literature search was conducted in the MEDLINE database using a Medical Subject Headings (MeSH) string combined with the Boolean operators “AND” and “OR.” The search terms for the first concept were as follows: “autoimmunity,” “autoimmune disease,” “autoimmune disorder,” and “self-reactive response” combined using “OR.” As for the second concept, the exploded MeSH term “bacteria” was used in combination with synonyms for bacteria, which were combined with the Boolean operator “OR” as follows: “acetobacter,” “Acetobacteraceae,” “Aerococcus,” “Bacillaceae,” “bacillus,” “corynebacterium,” and “Escherichia coli.” The third concept was set as for the second but using “AND” as follows: “lactic acid bacteria,” “Lactobacillaceae,” “Lactobacillus,” “probiotics,” “bifidobacteria,” and “bifidi.” Moreover, for the final concept, the exploded MeSH term “inflammatory response” was used. As for further concept combinations, we used, as explained above, “AND” or “OR.” We used the following major inclusion criteria (1): clinical trials (2) preclinical studies. The exclusion criteria were: (1) reviews (2) research involving prokaryotes (3) no relation to autoimmunity (4) no specific interest in intestinal flora (5) no relationship with probiotics (6) gene not belonging to the prokaryote (7) commentaries.

### 5.1 Inclusion and exclusion criteria

All studies met the following inclusion criteria: 1) studies had to be original research articles; review papers, case reports, or *in vitro* studies were initially excluded; 2) studies were required to report human primary outcomes related to the microbiome in autoimmune diseases; and 3) the studies had to use high-throughput sequencing to evaluate the composition and/or function of the microbiome. We conducted the literature search regardless of linguistic limitations. After initial exclusions and the subsequent review of the full text, we then evaluated whether the study contained baseline information about the autoimmune disease that was analyzed. Using this approach, the following diseases were selected: RA, T1D, G1D, SLE, and IBD. Then, we evaluated whether the autoimmune disease in question was diagnosed, and thereafter the progression of the effects on the microbiome was analyzed. In this step, studies that did not contain the following information were excluded: genus, family, or species levels; and relative abundance, absolute abundance from differences between the groups, or richness equal to or greater than 50% of the cuts through the taxonomic identification of bacteria (Ventelä et al., 2023).

### 5.2 Key findings from literature review

Based on the findings of this study, several novel aspects about gut microbiota dysbiosis associated with autoimmunity and inflammatory diseases were discussed. Based on the collated data, we suggest that autoimmune conditions share common features such as enrichment of specific taxa and genes but not groups of taxa. We posit that despite the need for sample size expansion and study verifications, the possible universal method for amelioration of autoimmunity could involve modification of the gut environment and promotion of gut health. To achieve such results, we foresee fundamental influences such as diet, living environment, hygienic conditions, and oral use of gut health-promoting probiotics, and lifestyle changes (McLean et al., 2014; Shaheen et al., 2022).

### 5.3 Systematic review results

The majority of the exponents in both datasets reported a decrease in microbiome diversity across all major immune-mediated diseases examined. The estimated diversity of microbiota across all immune-mediated diseases increases by 0.36 when absolute taxonomic richness is considered. Consistently, disease was associated with a 0.11 decrease, furthermore showing little heterogeneity. While no clear conclusions may be drawn from a funnel plot, the medium number of studies did not directly support any risk of a large number of experiments reporting smaller effects. Thus, careful interpretation of all results is appropriate; in particular, certain contributory factors regarding the intrinsic implications of the effect may need amendments to current standards in studies of immune-related diseases (Wang et al., 2023).

## 6 Implications for clinical practice

There are significant clinical implications for the study of the microbiome in relation to autoimmunity. Solutions to diseases like MS, lupus, and rheumatoid arthritis, diseases with a “common thread” for disease onset to understand and control, have thus far been elusive. Treatments such as T-cell therapy or stem cell therapy may be available in some countries, but have significant potential for serious side effects, including mortality. Probiotic therapy presents a paradigm shift in how we may treat diseases such as MS, lupus, and rheumatoid arthritis. These treatments have been described as being active, healthy, and inexpensive and positively contribute to a person’s health. There are well-documented outcomes in reducing infections, helping with obesity, and treating inflammatory bowel disease, all without significant negative side effects. In the context of autoimmunity, there is unique power in the potential ability to correct the problem at its very earliest cellular stages, prior to activation of innate and adaptive immune responses. Finally, the growing scientific support for the microbiome’s role in autoimmunity reinforces the notion that probiotic use can be rational to recommend, and that such recommendations can be embodied by guidelines that a physician can integrate into clinical practice (Bogović Crnčić et al., 2024).

### 6.1 Potential therapeutic strategies

Targeted gastrointestinal manipulation is an intriguing area. Dietary interventions can be an attractive strategy for these microbiome-targeted therapies, including prebiotics, probiotics, fecal microbiota transplantation, and/or synbiotics, as well as the incorporation of novel non-digestible carbohydrates, polyunsaturated fatty acids, and a variety of other nutrition-based intakes referred to as postbiotics and metabiotics. It is also well known that bacteria in co-culture tend to be more effective; this has paved the way for a viable combination and greater efficacy in therapeutics. Postbiotics such as short-chain fatty acids, melatonin, and indole derivatives can mitigate inflammation. To this end, a more comprehensive analysis has shown that the adjuvant therapy of melatonin in addition to traditional antidepressant therapy appears to be useful in improving depressive symptoms. Small molecules from certain bacteria reverse microbiota changes that contribute to obesity and metabolic disorders, suggesting that these molecules may be useful to mitigate the risk of developing depression in patients with obesity. Banana flakes mixed with lupin have shown a promising effect in regulating the gut microbiome and inflammation in animal studies. Gut microbiome-targeted therapeutics aim to correct the dysbiosis in the mucosa and restore the network of bacterial targeting and crosstalk between multiple immune-influencing cells, both innate and adaptive that dictate gut tolerance or enteric inflammation. Hence, such a therapy could represent a promising approach to prevent and/or control dysbiosis-induced systemic inflammation, which is considered to contribute to autoimmune and autoimmune-related conditions. Collectively, a large body of animal and human studies has suggested that microbial bioproducts can harness enteric immune system development and function and beneficially clear systemic inflammation, which can ultimately drive systemic autoimmune responses (Cresci et al., 2020).

### 6.2 Future research directions

Autoimmune diseases constitute a significant public health burden, particularly in Western societies. Information regarding genes, dietary and lifestyle factors, and the immune phenotype that are linked to the microbiome can provide a synthesis of the triggering mechanisms. Future research studies focusing on both set points of the immune phenotype and long-term probiotic administration are needed to gain a full understanding of the potential therapeutic interventions related to the human microbiome (Kim et al., 2023).

## 7 Conclusion and summary

Currently, studies focusing on the role of the microbiome in autoimmunity are predominantly described using animal models. Different methodologies that lack sufficient validity result in unsystematic data analysis. The diminished sample size utilization leads to a scattering of data distribution and robustness. We are aware of the limitations of human studies, including the potential risk of bias. Animal model studies can sometimes provide controlled measures of disease induction and resolution. The role of microbiome influences on autoimmunity is more elusive than for infectious diseases. At this stage, it is not possible to predict the accrual necessary to definitively show clinical benefits of specific microbiome manipulations. Basic and clinical experiments should be incorporated into evaluating impacts on the microbiome in the context of autoimmune states. Additional robust studies and more meta-analyses are required before the underlying pathological mechanisms of microbiome influence on autoimmunity are better elucidated. Such studies are still opening new avenues and have the potential to decrease inflammation through manipulation of the microbiome and may avert or delay the onset of autoimmunity. In summary, our analyses suggest that the role of the microbiome in autoimmunity is credible, and current evidence supports the development of strategies to modulate the variability in gut microbiota for the management of autoimmune diseases.

## Data Availability

The original contributions presented in the study are included in the article/supplementary material. Further inquiries can be directed to the corresponding author.
